# *Lactobacillus rhamnosus GG* Promotes Recovery of the Colon Barrier in Septic Mice through Accelerating ISCs Regeneration

**DOI:** 10.3390/nu15030672

**Published:** 2023-01-28

**Authors:** Lufang Chen, Shumin Li, Chunting Peng, Qifeng Gui, Jinyou Li, Zherong Xu, Yunmei Yang

**Affiliations:** 1 Department of Geriatrics, The First Affiliated Hospital, School of Medicine, Zhejiang University, Hangzhou 310003, China; 2 Key Laboratory of Diagnosis and Treatment of Aging and Physic-Chemical Injury Diseases of Zhejiang Province, The First Affiliated Hospital, School of Medicine, Zhejiang University, Hangzhou 310003, China

**Keywords:** *Lactobacillus rhamnosus GG* (*LGG*), sepsis, intestinal barrier dysfunction, intestinal stem cells (ISCs), colonoid, RNA-sequencing

## Abstract

Disruption of the intestinal barrier is both the cause and result of sepsis. The proliferation and differentiation of intestinal stem cells (ISCs) promote the regenerative nature of intestinal epithelial cells, repairing the injured intestinal mucosal barrier; however, it is uncertain whether the recovery effects mediated by the ISCs are related to the gut microbiota. This research found that the survival rate of septic mice was improved with a *Lactobacillus rhamnosus GG* (*LGG*) treatment. Furthermore, an increased proliferation and decreased apoptosis in colon epithelial cells were observed in the *LGG*-treated septic mice. In vitro, we found that a *LGG* supernatant was effective in maintaining the colonoid morphology and proliferation under the damage of TNF-α. Both in the mice colon and the colonoid, the *LGG*-induced barrier repair process was accompanied by an increased expression of Lgr5^+^ and lysozyme^+^ cells. This may be attributed to the upregulation of the IL-17, retinol metabolism, NF-kappa B and the MAPK signaling pathways, among which, Tnfaip3 and Nfkbia could be used as two potential biomarkers for *LGG* in intestinal inflammation therapy. In conclusion, our finding suggests that *LGG* protects a sepsis-injured intestinal barrier by promoting ISCs regeneration, highlighting the protective mechanism of oral probiotic consumption in sepsis.

## 1. Introduction

Intestinal epithelium dysfunction induced by sepsis results in an increased translocation of bacteria to the blood, contributing to the adverse outcome of sepsis [1]. The disturbance of the gut microbiota balance plays an essential role in intestinal mucosal barrier dysfunction, leading to the development of several highly prevalent diseases, including irritable bowel syndrome [2], inflammatory bowel disease [3] and sepsis [4]. Although no definite mechanisms have been elucidated that demonstrate intestinal microbiota alterations result in intestinal barrier dysfunction, more and more researchers have tried to modify the intestinal microbiota as a potential therapy to cure the diseases [5,6]. *Lactobacillus rhamnosus GG* (*LGG*) has been one of the most widely used probiotic genera to remold the intestinal microbiota; however, the effects of *LGG* on modulating the intestinal barrier function remain largely uncertain, and lacking an appropriate in vitro model has been one of the reasons.

Due to a series of host biological reactions, including cell proliferation and apoptosis, anti-oxidative stress, metabolism, and immune activity that can be affected by *LGG* [7], exploring the mechanisms by which *LGG* acts on traditional 2D intestinal epithelial cell cultures may be limited. Therefore, a 3D colonoid model composed of enterocyte cells, goblet cells, intestinal stem cells (ISCs), Paneth cells, and enteroendocrine cells [8] was used to study the effect of *LGG* on the intestinal barrier function. Colonoids are characterized by self-differentiation, organization, and forming spatial structures such as the intestine in vivo, making them an ideal model for the research of colon disease pathogenesis [9] and drug discovery [10].

This study combined colonoid and RNA-sequencing (RNA-seq) to investigate the effects of *LGG* on the intestinal barrier function in sepsis. Colonoids were applied to study whether a pretreatment with the supernatant of *LGG* can prevent TNF-α-induced changes in ISCs, Paneth cells, and tight junction protein expression. An RNA-seq analysis revealed the transcriptome profiling of colonoids with different treatments and this might help to obtain further insights about the beneficial effects of *LGG* [11]. Therefore, we next used the RNA-seq to recognize differentially expressed genes (DEGs) and gene co-expression networks that regulate ISCs regeneration responding to a TNF-α or *LGG* treatment. In summary, we established a new platform and explored the stimulatory effect of *LGG* on ISCs regeneration, which explains the protective effects of *LGG* on the colon barrier in another way, except for competing with pathogenic bacteria and producing antimicrobial peptides [7].

## 2. Material and Methods

### 2.1. Ethics Statement

Four-weeks-old male C57BL6 mice were purchased from the Experimental Animal Center of Zhejiang (license number: SCXK (J) 2019-0002). The animal research was approved by the Animal Experimental Ethical Inspection of the First Affiliated Hospital, College of Medicine, Zhejiang University and followed the guidelines of the American Association for Laboratory Animal Science.

### 2.2. Mice Treatment and Septic Model Building

The mice were split randomly into a LGG + septic group (where the mice were administrated intragastrically with 200 μL of *Lactobacillus rhamnosus GG* (ATCC 53103) (2 × 10^9^ CFU/mL) once a day for four weeks before a cecum ligation and puncture (CLP) operation), a control septic group, and a sham group (where the mice were administrated intragastrically with 200 μL of normal saline once a day for four weeks before a CLP or a sham operation), respectively. The detailed steps of the CLP operation refer to the research of Rittirsch D [12]. The mice in the sham group received the same procedures as the other two groups except for the CLP step.

### 2.3. Preparation of LGG Supernatant

The *LGG* was diluted in a MRS broth (Merck KGaA, Darmstadt, Land Hessen, Germany), and maintained on MRS agar plates (Merck KGaA, Darmstadt, Land Hessen, Germany) at 37 °C for 24 h. Bacterial 16S ribosomal RNA was sequenced to confirm the *LGG* strain. The confirmed *LGGs* were cultured in the MRS broth at 37 °C overnight until the logarithmic phase. The medium was centrifuged (at 8000 rpm, for 15 min, at 4 °C), and the supernatant was filtered with a 0.2 μm filter to remove the live bacteria. *Escherichia coli* MG1655 (ATCC 700926) were cultured in a brain heart infusion (BHI) broth as appropriate at 37 °C under aerobic conditions overnight until reaching the logarithmic phase. The supernatant was then obtained just as in the steps for the *LGG*.

### 2.4. Enteroids Establishment and Maintenance

Stem cell-enriched proximal colon fractions from four-week-old C57BL6 male mice were used to culture the colonoids. Briefly, the proximal 3–6 cm of the colon was obtained and washed with cold Dulbecco’s Phosphate buffered-saline without Ca^2+^ and Mg^2+^ (DPBS) (Gibco, Waltham, MA, USA) three times. Then, a vertical incision was made in the colon fractions, gently rewashed using DPBS several times, and cut into 2 mm pieces. To isolate the crypts from the basal membrane, the colon pieces were incubated in a Gentle Cell Dissociation Reagent (GCDR) (Stemcell Technologies Inc., Vancouver, BC, Canada) for 20 min at room temperature, centrifuged at 20 rpm, then resuspended with cold DPBS containing 0.1% bovine serum albumin (BSA) (Sigma-Aldrich, St. Louis, MO, USA), before being stood for 30 s until most of the colon fractions were sinking to the bottom. The supernatants were filtered through a 70 μm cell mesh (Corning Costar Corp, Corning, NY, USA) and collected in a 50 mL centrifuge tube. The resuspension and filtration steps were repeated three times to obtain the other three supernatants, choosing one that contained the most crypts for the following colonoids culture. After counting under light microscopy, the colon crypts were resuspended using a mixture of IntestiCult™ Organoid Growth Medium (Stem cell Technologies Inc., Vancouver, Canada) and Matrigel Matrix (Corning Costar Corp, Corning, NY, USA) in a ratio of 1:1. Then, 50 µL of the colon crypt suspension (500 crypts) in each well was planted on a prewarmed 24-well plate to form a dome, and 750 μL of room-temperature Organoid Growth Medium was added to each well and kept at 37 °C. The medium was changed three times per week after the enteroids were established, and the colonoids were passaged every 7–10 days in a ratio of 1:2.

### 2.5. Enteroids Treated with TNF-α and Pre-Treated with the Supernatant of LGG

The colonoids were divided into four groups. The first, was a TNF-α group (where the mice colonoids were treated with TNF-α (100  ng/mL) for 24 h). Second, was a LGG + TNF-α group (where the mice colonoids were treated with TNF-α (100  ng/mL) for 24 h, but before that, the colonoids were co-cultured with the supernatant of LGG (5 μL per well) for 12 h). The supernatant of the LGG addition promoted the growth of colonoids in a dose-dependent manner; therefore, 5 μL per well of the LGG supernatant was chosen as the test dose in subsequent experiments. Third, was an *E. coli* + TNF-α group (where the mice colonoids were treated with TNF-α (100  ng/mL) for 24 h, but before that, the colonoids were co-cultured with a supernatant of *E. coli* (5 μL per well) for 12 h). Here, both live and heat-killed LGG decreased LPS-induced proinflammatory mediators and increased anti-inflammatory mediators [13]. Moreover, the metabolites of the LGG were high-temperature resistant; therefore, we used the culture supernatant of Escherichia coli MG1655 (ATCC 700926) as the bacteria control. Finally, there was a control group (where the mice colonoids were cultured with an Organoid Growth Medium added with BHI. The cell viability was evaluated using the Cell Titer-GLO cell viability kit (Promega, Madison, WI, USA) according to the protocol of the manufacturer. The luminescence was measured using the Glomax Multi Plus Detection System (Promega, Madison, WI, USA). The intensity of the luminescence signal was proportional to the number of the ATP, which correlated positively with cell viability. In this research, there was only one batch of LGG metabolites used. The metabolites of the LGG mainly consisted of lactic acid, acetate, butyrate and propionate. All these metabolites can be analyzed by gas chromatography mass spectrometry (GC/MS). Therefore, if more than one batch of the LGG metabolites will be used in further research, GC/MS could be used to quantify the main metabolites of the LGG so that different preparations can be unified.

### 2.6. Histological Analysis

For mice tissue staining, the proximal colon tissues of the C57BL6 mice were fixed in 4% paraformaldehyde (Sigma-Aldrich, St. Louis, MO, USA) for 24 h, then embedded in paraffin, and sectioned at 4 μm. For the colonoids staining, the colonoids were obtained and fixed in 4% paraformaldehyde at 4 °C for 6 h, then dehydrated with graded ethanol, embedded in paraffin and cut at 0.5 μm for immunostaining. The sections of the colon tissue and colonoids were deparaffinized and hydrated, and then incubated with anti-mouse occludin antibody (1:200, ab216327, Abcam, Cambridge, MA, USA), anti-mouse lysozyme antibody (1:100, ab108508, Abcam, Cambridge, MA, USA), anti-mouse Lgr5 antibody (1:100, Clone-OTI2A2, OriGene Technologies, Rockville, MD, USA), or anti-mouse Ki67 antibody (1:200, ab15580, Abcam, Cambridge, MA, USA) overnight. Subsequently, the sections were incubated with secondary antibodies (1:200, ab150117, Abcam, Cambridge, MA, USA) for 1 h at room temperature. Moreover, the samples were treated with a terminal deoxynucleotidyl-transferase-mediated dUTP-biotin nick end-labeling (TUNEL) (Invitrogen Corp, Carlsbad, CA, USA) assay to evaluate the apoptosis of both the colon epithelial cells and colonoids.

### 2.7. LGG Detection in Fecal Samples

Fecal samples were obtained from a separate set of mice before the CLP operation (control, *n* = 5; *LGG*, *n* = 5). The DNA was extracted from frozen fecal samples using a QiaAMP DNA stool Minikit (Qiagen, Valencia, CA) according to the manufacturer’s instructions. The *LGG* quantification was performed using a quantitative PCR (QT-PCR) with the following primers: LactoF, 5′-AGCAGTAGGGAATCTTCCA-3′; and LactoR, 5′-ATTYCACCGCTACACATG-3′. The PCR reaction was performed as follows: at 95 °C for 5 min, followed by 35 cycles at 94 °C for 15 s, then at 53 °C for 30 s, and at 72 °C for 45 s before a final extension at 72 °C for 10 min. The quantification data from the PCR analysis were expressed as log qPCR copy/fecal (g).

### 2.8. Quantitative PCR

The total RNA from the colon tissue and colonoids was extracted by using TRIzol (Invitrogen Corp, Carlsbad, CA, USA), and then subjected to a reverse-transcribed reaction with a miScript Reverse Transcription kit (Qiagen GmbH, Hilden, Germany). The synthesized cDNA from the above steps was applied for the quantitative PCR (QT-PCR) using SYBR-Green (Takara Bio, Tokyo, Japan). Table 1 shows the primers for the *Occluding*, *Lgr*5, *Ascl*2, *Olfm*4, *Lyz*1, *Defa*6, *Tnfaip3*, *Nfkbia*, and *GAPDH*.

### 2.9. RNA-Sequence

Cellular RNA was extracted from the colonoids and 4 μg of RNA was extracted from the control, the TNF-α group and the *LGG* + TNF-α group of murine colonoids (with three replicates of each group). They were then submitted for a subsequent library construction. The RNA-seq libraries were established applying a NEBNext Ultra™ RNA Library Prep Kit for Illumina (New England BioLabs, Ipswich, MA, USA) according to the operation manual of the manufacturer, and the index codes were introduced to identify the different sequencing samples. After constructing the libraries, a Qubit2.0 Fluorometer was used for the initial quantification, and then an Agilent 2100 bioanalyzer (Agilent Technologies, Palo Alto, Calif) was applied to test the insert size of the library; when the latter met expectations, a qRT-PCR was next adopted to quantify the effective concentration of the library. An effective concentration ≥ 2 nM was considered as a qualified library. After the quality test, the libraries were pooled according to the effective concentration and demand for targeting offline data volume, and then sequenced on an Illumina Hiseq platform. Raw data was presented in the form of a fastq format, and was filtered by removing reads with an adapter, namely, reads containing N (i.e., unable to determine the base information), and low-quality bases to obtain clean reads. Hisat2 v2.0.5 (http://ccb.jhu.edu/software/hisat2/index.shtml, accessed on 8 October 2022) was used to compare the clean reads with the reference genome to obtain the location information of the reads in the reference genome. The read numbers mapped to each gene were calculated using the Htseq-count package. Then, the fragments per kilobase million (FPKM) of each gene was obtained based on the depth and gene length of the sequencing. DEGs between the TNF-α group and the *LGG* + TNF-α group were determined using the DESeq2 R package. A significance analysis of the microarray data was performed, with the selection criteria as follows: (1) an adjusted *p*-value (*P*adj) ≤ 0.05; and (2) |log2 (fold change)| >  1. The genes were classified into up-regulated and down-regulated genes based on the |log2 (fold change)| value. The selected DEGs were further used for the GO (Gene Ontology) database and a KEGG (Kyoto Encyclopedia of Genes and Genomes) analysis. The GO is a database developed to describe gene functions. The KEGG is a database that integrates information on genomic, chemical and system functions. The R packages of the “top GO” and “Cluster Profiler” were used for the GO and KEGG analyses, respectively. *P*adj ≤ 0.05 was a statistical difference in the GO and KEGG analyses. Finally, Cytoscape (version 3.9.1) was applied to score the effect of different genes on the protein–protein interaction (PPI) network and to generate an *LGG* pathway target interaction network to identify the hub genes.

### 2.10. Statistical Analysis

GraphPad Prism 6.0 (GraphPad Software Inc., San Diego, CA, USA) was used for the data analysis. The Student’s *t* test was applied to compare the abundance of *LGG* in the fecal segment of the colon. A one-way analysis of variance (ANOVA) with a post hoc Bonferroni test was used to compare the variables of the gene expression, tissues, and colonoids among the three groups. The data were presented as means ± SD, and a *p* ≤ 0.05 was considered statistically significant.

## 3. Results

### 3.1. Colonization of LGG on Colon Mucosa

**Figure 1:**
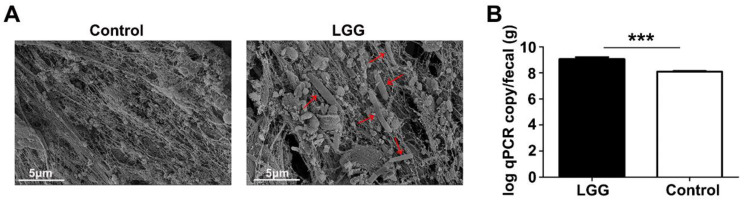
Colonization of *LGG* in the colon mucosa. (**A**) Scanning electron microscopy shows that *LGG* (red arrow) can engraft in colon mucosa after intragastric administration for four weeks; (**B**) quantification data of fecal *LGG* from the qt-PCR analysis are expressed as log qPCR copy/fecal (g). *** *p <* 0.001.

Scanning electron microscopy was first used to assess the colonization effects of LGG to observe the number of bacteria engrafted in the colon mucosa. After an intragastric administration of *LGG* for four weeks, the *LGG* colonized in the mucosa of the colon under an electron microscope, which was essential for the *LGG* to interact with the colon epithelium (Figure 1A). A PCR was further used to quantify the abundance of *LGG* in the fecal colon segment. A statistical increase in the *LGG* abundance was observed in the mice fed with *LGG* (Figure 1B). The above data suggests that *LGG* can effectively pass through the gastrointestinal tract and colonize the colon epithelium after an oral administration.

### 3.2. LGG Ameliorates Intestinal Barrier Injury in Septic Mice

**Figure 2:**
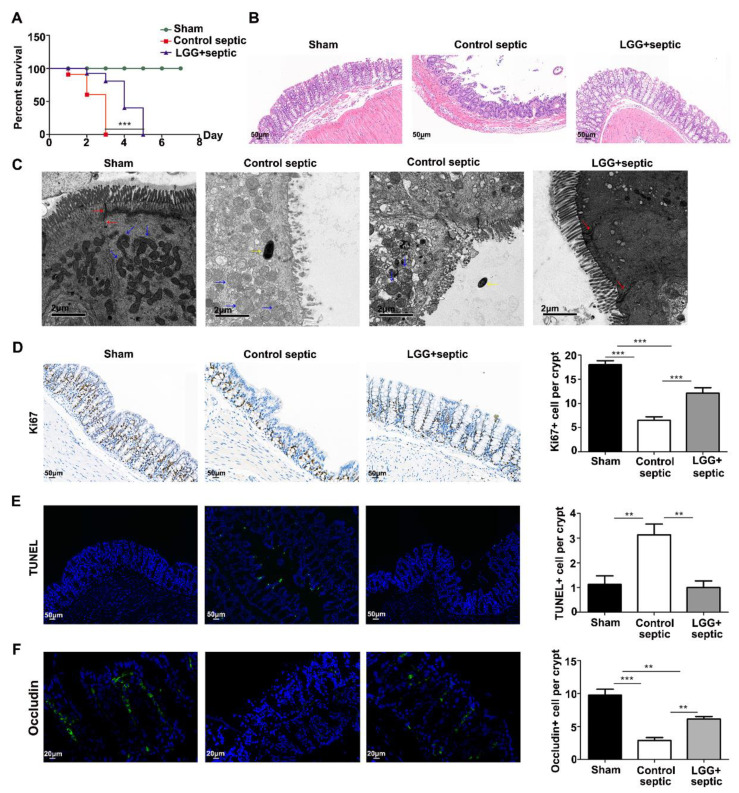
*Prophylactic LGG* treatment alleviates mucosal barrier injury in septic mice. (**A**) Kaplan–Meier survival curve after CLP operation; *LGG* significantly decreases the mortality rate in septic mice; all sham mice survived (*n* = 5 per group); (**B**) H&E staining of colon sections from the sham group (left), control septic group (middle), and *LGG*-pre-treated septic group (right), respectively; (**C**) transmission electron microscopy shows the morphology of a colon gland: tight connection (red arrow), mitochondria (blue arrow), and bacteria invasion (yellow arrow) in the sham group (left), control septic group (middle), and *LGG* pre-treated septic group (right); (**D**) Ki67 assesses proliferation, and (**E**) TUNEL assesses apoptosis: staining of colon sections from the sham group (left), control septic group (middle), and *LGG*-pre-treated septic group (right), respectively; (**F**) occludin staining of colon sections from the sham: control-septic, and *LGG* + septic groups (*n* = 6 per group from (**B**–**F**)). ** *p*< 0.01; *** *p <* 0.001.

CLP mice were a classical model to study sepsis. To evaluate the effects of *LGG* on sepsis, four-week-old male C57BL/6 mice were administered orally with *LGG* or normal saline for four weeks, then subjected to a CLP operation to establish a sepsis model. The mortality rate in the LGG stomach perfusion septic-mice group decreased markedly compared to the control-septic group (Figure 2A). Furthermore, sepsis significantly damaged the integrity of the colon epithelial structure, while the *LGG* minimized this destruction of the colon epithelial barrier caused by the sepsis. A histological examination showed a more extensive gland deformation (Figure 2B) and a reduction in tight connections (Figure 2F) in the colonic tissues of the control-septic mice. On the contrary, a tight connection loss, bacteria invasion, and mitochondrial swelling were observed under transmission electron microscopy in the control group of septic mice (Figure 2C). The pathological injury, however, induced by the sepsis was ameliorated in the *LGG*-pretreated septic group, which generally showed a regular colonic structure and ultrastructure using a light and transmission electron microscopic (Figure 2B,C).

The protective effect of the *LGG* was also reflected in the proliferation activity and apoptotic status of the colon epithelial cells. Sepsis destroyed the colon morphology with a lower expression of Ki67-positive cells and higher TUNEL-positive cells; however, the *LGG* significantly increased the Ki-67 positive rate. In contrast, it decreased the TUNEL-positive rate caused by the sepsis (Figure 2D,E), suggesting that *LGG* promotes the epithelial barrier recovery process by stimulating proliferation and inhibiting the apoptotic status in sepsis progression.

### 3.3. LGG Promotes Growth of Colonoids and Recovery from TNF-α Injury

**Figure 3:**
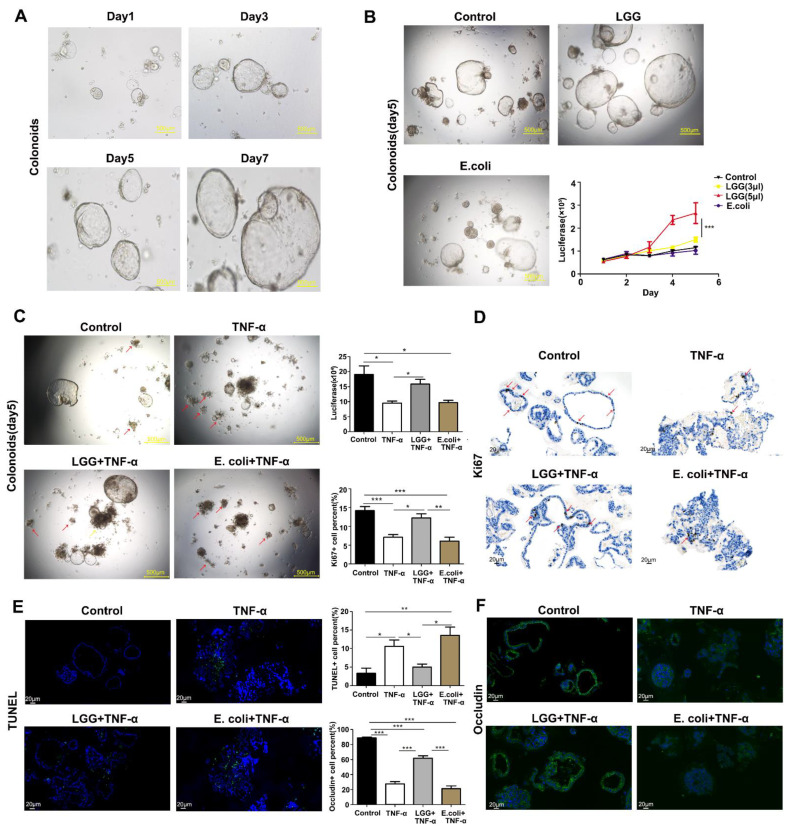
*LGG* promotes the recovery of colonoids after damage by TNF-α. (**A**) The growth status of colonoids on days 1, 3, 5, and 7 under a light microscope. Scale bar: 500 μm; (**B**) the growth status of colonoids treated with the supernatant of *LGG* and with the supernatant of *E. coli* strain MG1655 (bacteria control) with 5 μL per well on day 5 (left panel) and the viability of colonoids analyzed using the Cell Titer-GLO assay (right panel), with *n =* 3 wells per group. Scale bar: 500 μm; (**C**) colonoids treated with TNF-α (100 ng/mL) for 24 h with *LGG* supernatant and *E. coli* supernatant (5 µL per well) for a 12 h intervention. The morphology of the colonoids on day 5 was observed under a light microscope and the damaged colonoids (black and shrunken in morphology) are indicated with a red arrow, while the budding colonoids are shown with a yellow arrow, and the colonoid viability is presented with a luminescence according to the CTG assay with *n =* 3 wells per group. Scale bar: 500 μm; (**D**) Ki67 staining of colonoids from the control group, TNF-α group, *LGG* + TNF-α group, and *E. coli* + TNF-α group, respectively. Ki67-positive granules are indicated with a red arrow with *n =* 30 colonoids per group. Scale bar: 20 μm. (**E**) TUNEL staining of colonoids from the control group, TNF-α group, *LGG*+ TNF-α group, and *E. coli* + TNF-α group, respectively, with *n* = 30 colonoids per group. Scale bar: 20 μm. (**F**) Occludin staining (green fluorescence) for a tight connection in colonoids from the control, TNF-α, *LGG* + TNF-α, and *E. coli* + TNF-α groups, respectively, with *n =* 30 colonoids per group. Scale bar: 20 μm. * *p <* 0.05, ** *p <* 0.01, *** *p <* 0.001.

To further explore the potential mechanism of *LGG* in vitro, a mouse colonoid model was used to assess whether the LGG supernatant (mainly containing metabolites of *LGG*) could protect the colonoids from the damage caused by the TNF-inflammatory factor TNF-α damage. First, we established a mice colonoid model (Figure 3A) and observed that an *LGG* addition promoted the growth of colonoids in a dose-dependent manner. Therefore, 5 µL per well of the *LGG* supernatant was chosen as the test dose in the subsequent experiments (Figure 3B). Consequently, we found that the colonoids in the control group grew well with clearer crypts of the growth, larger surface areas and an increasing number of colonoids of the growth (yellow arrow) compared to the TNF-α-treated group. After the treatment of TNF-α, the number of damaged colonoids grew, and the latter was characterized by a black and shrunken appearance (red arrows) and a budding reduction (Figure 3C). Moreover, the TNF-α injured the energy metabolism of the cells and reduced the cell activity by decreasing the level of ATP (Figure 3C); however, the extent of the colonoid damage and TNF-induced cell viability injury induced by the TNF-α was markedly alleviated by the *LGG* intervention. Conversely, the *E. coli* treatment had no protective effect on the colonoid damage.

To evaluate the growth-promoting effects of *LGG* on colonoids, a Ki67 staining was used to measure the proliferation, while a TUNEL assay was applied to assess the apoptosis. The TNF-α caused a few Ki67-positive and more TUNEL-positive cells in the crypt of colonoids than in the control group; however, the *LGG* statistically increased the number of Ki67-positive cells and decreased the ratio of TUNEL-positive cells, consistent with keeping the normal morphology of colonoids and reducing the number of injured colonoids (Figure 3D). This suggests that *LGG* quickens the recovery process in an inflammatory injury by stimulating the proliferation and inhibiting the apoptosis of epithelial cells. Conversely, the *E. coli* supernatant did not show a stimulatory effect on the epithelial proliferation.

### 3.4. LGG Promotes the Activation and Proliferation of ISCs after Damage

**Figure 4:**
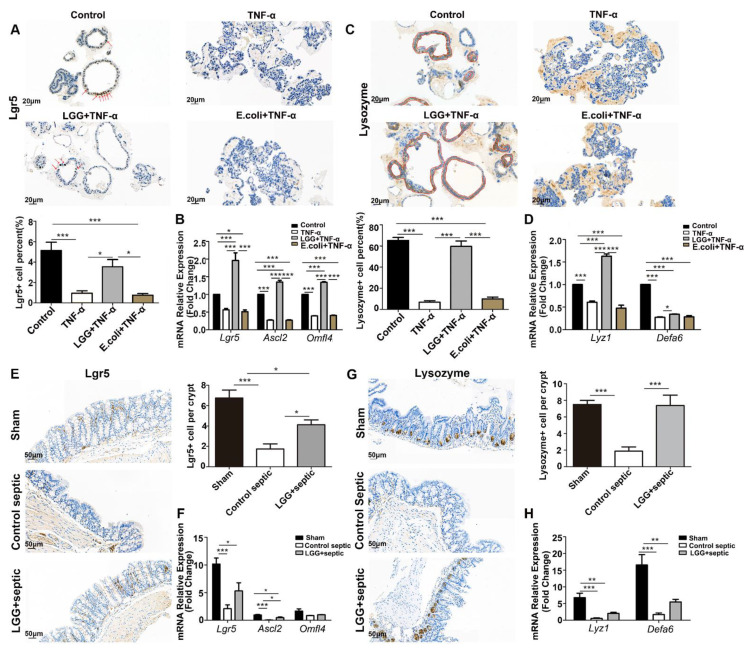
*LGG* stimulates the regeneration of ISCs. (**A**) Lgr5 staining of colonoids in the control group, TNF-α group, *LGG* (supernatant of *LGG* (5 μL per well)) + TNF-α group, and *E. coli* (5 μL per well) + TNF-α group. The number of Lgr5^+^ cells (red arrows) was then counted with *n =* 30 colonoids per group. Scale bar: 20 μm. (**B**,**D**) Relative expression of ISCs mRNA (*Lgr5*, *Ascl2*, and *Olfm4*) and Paneth cells mRNA (*Lyz1* and *Defa6*) of colonoids in the control, TNF-α, and *LGG*+ TNF-α groups, and the *E. coli* + TNF-α group were quantified using qt-PCR with *n =* 3 wells per group. (**C**) Lysozyme staining of colonoids in four groups; red lines indicate lysozyme^+^ cells with *n =* 30 colonoids per group. Scale bar: 20 μm. (**E**,**G**) IHC images (brown granules in the nucleus of endothelial cells are Lgr5-positive cells, while brown granules in cytoplasm indicate lysozyme-positive cells (Paneth cells)) of the colon with different treatments, and the number of Lgr5- and lysozyme-positive cells were counted. Scale bar: 50 μm. (**F**,**H**) Relative expression of ISCs mRNA (*Lgr5*, *Ascl2*, and *Olfm4*) and Paneth cells mRNA (*Lyz1* and *Defa6*) of colon tissue in the sham, control-septic, and *LGG*+ septic groups quantified using qt-PCR, respectively, with *n* = 6 per group. * *p <* 0.05, ** *p <* 0.01, *** *p <* 0.001.

Regenerating the ISCs plays an important role in repairing colon epithelial damage. Accordingly, we evaluated the expression of Lgr5-positive and lysozyme-positive cells in the crypt of colonoids characterized by secreting stem niche factors, including Wnt3, EGF, and TGF-α, to maintain the microenvironment of the ISCs, and to modulate the proliferation and differentiation of the ISCs. We found a significantly lower proportion of Lgr5-positive cells in colonoids damaged by TNF-α, accompanied by a decreased expression of Lgr5, Ascl2, and Olfm4 (the markers of ISCs); however, the declining trend was reversed by the LGG instead of the *E. coli* treatment (Figure 4A–B). Furthermore, the LGG rather than the *E. coli* increased the lysozyme-positive cells and expression of Lyz1 and Defa6 (i.e., the markers of Paneth cells) in the TNF-α-treated colonoids (Figure 4C–D). The same stimulatory effect of LGG on the ISCs and lysozyme-positive cells was also observed in the septic mice (Figure 4E–H). Unlike the small intestine, Lgr5-positive cells are usually located deep in the crypt, while in the large intestine (mainly the colon), Lgr5-positive cells cannot re-locate towards the base of the crypt and so are lost from the niche relatively quickly; therefore, Lgr5-positive cells can locate far from the base of the crypt [14]. In our results, Lgr5-expressing cells located both deep in the crypt and far from the base of the crypt were decreased in the colon of the septic mice; however, the downward trend was reversed with the LGG treatment (Figure 4E). Paneth cells are normally present in the human cecum and ascending colon, but are rarely found in the descending colon and rectum. Paneth cell metaplasia and aberrant lysozyme production in the descending colon and rectum are hallmarks of IBD pathology. In our study, both a proximal and distal colon segment were obtained and stained with lysozyme; however, lysozyme-positive cells were not seen in the distal colon, but they were present in the proximal colon just beside the cecum (similar as in humans). According to Klaus Lewin’s research [15], in Crohn’s disease, a decrease in the number of Paneth cells occurred in severely diseased segments. In less acutely-inflamed specimens and in areas where there was evidence of repair, a proliferation of Paneth cells was seen. Here, the colon injury and inflammatory reaction caused by the CLP model was very acute and severe; therefore, the number of Paneth cells was significantly decreased in the septic mice group, whereas, a proliferation of Paneth cells was seen in the LGG- treated septic group as the evidence of repair (Figure 4G).

### 3.5. LGG Pretreatment Remolds the Transcriptional Profile in TNF-Injured Colonoids Injured by TNF-α

**Figure 5:**
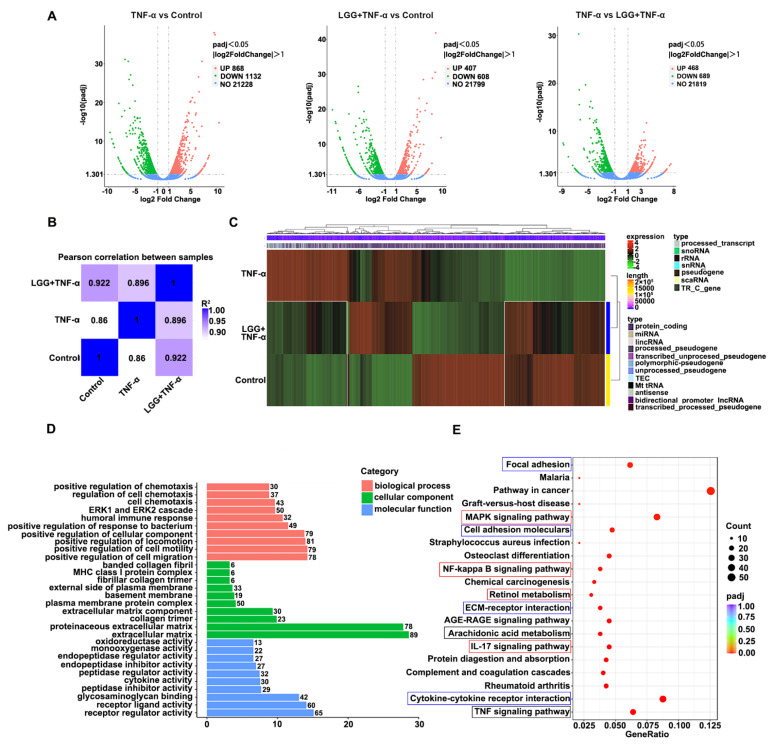
*LGG* alters the transcriptional profile in colonoids after TNF-α injury. (**A**) The volcano plot shows DEGs in colonoids among the control, TNF-α, and *LGG* + TNF-α groups using a RNA-seq; (**B**) Pearson’s correlation analysis between the three groups (where the closer the value of R^2^ is to 1, the higher the value of relevance); (**C**) heat map of differential genes within the three groups, where after the intervention of *LGG*, the transcriptional profile in the TNF-α group became closer to that of the control group (white square); (**D**) GO analysis showing DEGs in biological processes, cellular components, and molecular functions, respectively; and (**E**) KEGG pathway of DEGs, where dots indicate the number of DEGs (the color of the dot represents the *P*adj value of the DEGs), and the red, blue, and black boxes represent the pathways related to ISCs’ regeneration, cell adhesion, cell apoptosis, and inflammation.

To further explore the protective mechanisms of *LGG*, we built a colonoid model to mimic IEC-microbe interactions in vitro. An RNA-seq analysis of the total RNA extracted from the colonoid stimulated with TNF-α, *LGG* + TNF-α, or just a colonoid growth medium was performed. We found 2000 DEGs between the TNF-α treated and control colonoid groups, with 868 upregulated, and 1132 downregulated in the TNF-α group compared to the control group. With the *LGG* treatment, the TNF-α only upregulated the expression of 407 genes of 1015 DEGs while decreasing 608 gene expressions compared to the control group (Figure 5A). Combined with the Pearson correlation heatmap between the samples, the correlation coefficient between the *LGG* + TNF-α and the control group (0.922) was markedly higher than that between the TNF-α and the control groups (0.86), indicating that the TNF-α induced a special gene transcriptional profile in the colonoids, which could be remolded by the *LGG* treatment (Figure 5B). Then, the differential genes of all three groups were obtained and collected as the differential gene set to form a clustering analysis heatmap. The DEGs and hierarchical clustering of the samples showed a completely differential expression between the TNF-α and the control groups; however, after the *LGG* treatment, the number of DEGs declined, and the transcriptional gene profile became more similar to the control group (white boxes) (Figure 5C). To further detect functional changes in the gene profiles caused by the TNF-α and *LGG* treatments, a GO analysis was applied between the *LGG* + TNF-α and TNF-α groups. The GO analysis showed that most DEGs induced by the *LGG* treatment in the biological process category were enriched in positive cell migration and chemotaxis regulation. In contrast, in the category of cellular components, the *LGG* affected mainly the extracellular matrix, the plasma membrane, and the basement membrane. Furthermore, several DEGs in the molecular function category were associated with cytokine activity and receptor regulator activity (Figure 5D).

A KEGG pathway analysis revealed that the DEGs caused by the TNF-α and *LGG* were mainly enriched in the IL-17-signaling pathway, retinol metabolism-signaling pathway, NF-kappa B-signaling pathway, and the MAPK-signaling pathway (red boxes). All of these signaling pathways were associated with the proliferation and regeneration of ISCs. Furthermore, the KEGG pathways involved in reconstructing the colon barrier, such as cell adhesion molecules, focal adhesion, ECM receptor interaction, cytokine–cytokine receptor interaction (blue boxes), and pathways related to cell apoptosis and inflammation—including the TNF-signaling pathway, arachidonic acid metabolism (black boxes)—were included in the top 20 pathways (Figure 5E). The above results suggest that the *LGG* maintenance of the proliferative potency of colon epithelium in colonoids may have arisen from the protection and promotion of ISCs’ renewal in the TNF-α group.

### 3.6. Construction of LGG-Pathway-Target Network and Module Analysis

**Figure 6:**
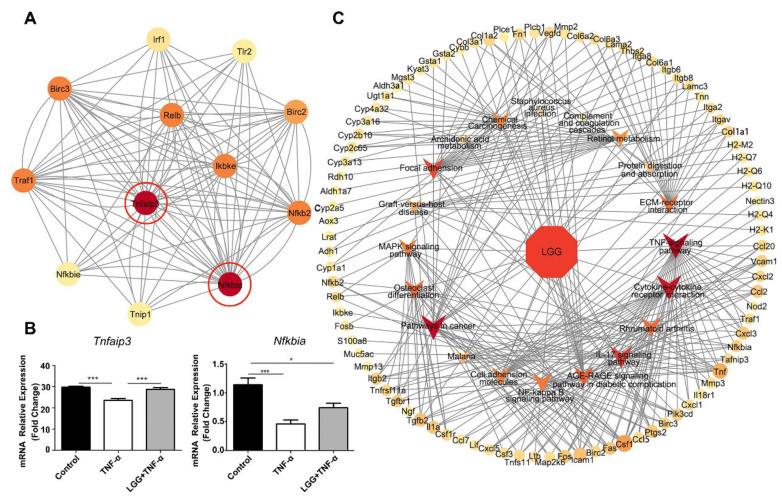
Construction of the *LGG*-pathway-target interaction network and module analysis. (**A**) Molecular complex detection (MOCDE) was used to find the key subnetworks and core genes among the PPI network; an MCODE score 5 was considered a significant module; panel A shows the top module with an MCODE score of 10.36; the two core genes in this group are colored in red; the higher the degree of relevance between other genes, the darker the color of the nodes is; (**B**) relative expression of *Tnfaip3* and *Nfkbia* of colonoids in the control, TNF-α, and *LGG*+ TNF-α groups were quantified using qRT-PCR with *n =* 3 wells per group; (**C**) the *LGG-*pathway-target interaction network was constructed using the Cytoscape software; the colored arrow indicates the 20 main pathways; the greater the number of genes in the pathway, the larger the size of the representative arrow is; the higher the relevance between other genes, the darker the color of the arrow and the node is. * *p <* 0.05, *** *p <* 0.001.

A PPI analysis of the DEGs revealed 286 nodes and 1355 interactions between the *LGG* + TNF-α and TNF-α groups. Using the MCODE in Cytoscape 3.9.1, the best modules of the PPI network were selected (Figure 6A), including *Nfkbia* and *Tnfaip3* as two hub genes for a high degree of connectivity. Both of these hub genes were downregulated in the TNF-α-treated colonoids. *LGG* was sufficient to prevent the downregulation of *Tnfaip3* and *Nfkbia* in the colonoids treated with TNF-α (Figure 6B). To illustrate the relationship between *LGG* and a TNF-α injury, an *LGG*-pathway-target interaction network was constructed through Cytoscape. The network consisted of *LGG*, the top 20 pathways related to the *LGG* treatment, and the common targets, which directly showed a multi-target effect of *LGG* in protecting the pathogenesis of a TNF-α injury, including the TNF-signaling pathway, cytokine–cytokine receptor interaction, focal adhesion, IL-17-signaling pathway, retinol metabolism-signaling pathway, NF-kappa B-signaling pathway, and MAPK-signaling pathway, that contributed the most (Figure 6C).

## 4. Discussion

Intestinal barrier dysfunction is one of the major health complications, increasing septic-patient mortality [1]. LGG, as a probiotic, is widely used in food additives and clinical research to prevent and cure some intestinal inflammatory diseases, due to its ability to protect the integrity of the intestinal barrier [16,17]. Several mechanisms may explain the beneficial effects of LGG, such as the encoding of a genome that synthesizes SpaCBA pili that play an important role in promoting biofilm formation to protect the mucosa mechanically [18], the inhibition of the formation of pathogen biofilms through LIp1(lectin-like protein 1) and Llp2 [19], the reduction in proinflammatory cytokine expression through a releasing of extracellular vesicles [20] and the production of peptides to exhibit antibacterial activities [21]. Murphey reported that immune function after CLP-induced sepsis was due to an exposure to microbial ligands within the cecal lumen rather than from tissue trauma, ischemia, or necrosis. This means that microbiota changes play an important role in the prognosis of sepsis [22]. In our previous study [23], mice were fed either probiotic LGG or saline four weeks before a CLP operation and fecal samples were collected and analyzed using 16S rDNA sequencing. We found that an LGG treatment can noticeably reduce sepsis mortality and reverse intestinal microbiota dysbiosis caused by sepsis. Besides remolding the microbiota, LGG was also found to repair colon barrier destruction that was induced by sepsis effectively, which was reflected in a stimulation of proliferation and an inhibition of apoptosis of colon epithelial cells, manifested in an increasing number of Lgr5-positive and lysozyme-positive cells. Additionally, Lgr5+ ISCs may differentiate into all cell types of the colon epithelium. In contrast, lysozyme-positive Paneth cells were found to provide an epithelial niche and to maintain a micro-environment for ISC growth and differentiation through the Wnt- and Notch-signaling pathways [24]. Therefore, we suppose that the protective effect of LGG may be related to regulating ISC self-renewal. 

To further illustrate the relationship between LGG and ISCs, the transcriptional profile in the colonoids treated with LGG + TNF-α or TNF-α only was obtained using a RNA-sequencing analysis. A GO analysis revealed that the positive regulation of cell migration and locomotion in the biological process category, in the extracellular matrix, the plasma membrane, and the basement membrane in the cellular component category, may be in-volved in the intervention of LGG in a TNF-α injury. In a steady state, ISCs are located at the base of the crypt and gradually migrate upward to a transit amplifying (TA) zone to experience a proliferation boom. Afterward, they continue to move to the top of the crypt and differentiate into all types of colon cells [25]. Following a TNF-α exposure, increased apoptosis and rapidly diminished proliferative compartments in the colon crypts caused the appearance of shrinkage of the colonoid; however, the trend of rapid loss was reversed by a burst of proliferation in the surviving Lgr5+ ISCs with the LGG treatment. Therefore, the positive regulation of cell migration and the locomotion in the GO analysis were the indicators of the ISCs’ proliferation and differentiation promoted by the LGG. 

The Wnt- and Notch-signaling pathways are critical for the proliferation and self-renewal of ISCs [26,27]. However, in our KEGG analysis and LGG-pathway-target interaction network, the IL-17-signaling pathway, retinol metabolism-signaling pathway, NF-kappa B-signaling pathway, and the MAPK-signaling pathway were shown to be the main contributors to LGG action. Produced by Th17 cells, Interleukin (IL)-17A promotes Atoh1 expression in Lgr5+ ISCs after injury, and Interleukin (IL)-17A leads to differentiation of ISCs into secretory cells; however, the recovery ability after intestinal injury is attenuated when IL-17RA is stowed away in ATOH1+ cells, suggesting that the IL-17-signaling pathway plays an important role in the regulation of Lgr5 + ISCs during injury responses [28]. As an alcohol form of vitamin A, retinol (ROL) can be metabolized to retinoic acid (RA), and both ROL and RA provide early signals for events cascade initiation to promote ISCs differentiation [29]. In addition to its important role in tumor formation, the NF-kappa B-signaling pathway is involved in maintaining intestinal epithelial homeostasis [30]. For example, NF-κB activity was found in the Paneth cells and Lgr5+ ISCs of small intestinal crypts. Moreover, deleting NF-κB causes a significant decrease in Paneth cells and increases the number of immature intermediate cells and goblet cells, indicating that the NF-kappa B-signaling pathway is essential for the maintenance of ISC niches by controlling the Paneth cell differentiation from secretory progenitor cells [31]. Wnt-signaling keeps the proliferation and stemness of ISCs, while little is known about the inhibitory effect of Wnt on ISCs. Zahra et al. [32] reported that suppressed Wnt-signaling can induce proliferation and MAPK-signaling in ISCs, indicating that Wnt-signaling maintains ISC pools by inhibiting the MAPK-signaling pathway, which then promotes the differentiation of ISCs into TA cells. Our KEGG analysis showed that the pathways which might be involved in the intervention of LGG in colonoid damage had a close relationship with ISCs proliferation and differentiation, and that LGG may interact with ISCs through multiple biological processes and signaling pathways.

In the PPI analysis, using the MCODE in Cytoscape, Tnfaip3 and Nfkbia were chosen as two hub genes for a high degree of connectivity. Tnfaip3, known as A20, maintains the stability of the intestinal barrier from IEC apoptosis caused by cytokines [33]. Mice with enterocyte- and myeloid cell-specific A20 knockout have an impaired barrier function at-tributed to the loss of goblet and Paneth cells, subsequently leading to a reduction in mucus and antimicrobial peptides, which then facilitates bacterial translocation. An A20 deletion in IECs, for example, sensitized the apoptosis of Paneth cells in cytokine exposure instead of causing Paneth cell death directly [34]. Herein, the LGG-treated colonoids presented an upregulated A20 expression and number of Paneth cells compared to colonoids treated with TNF-α only, suggesting that A20 may be used as a potential biomarker for LGG in the therapy of intestinal inflammation. Besides Tnfaip3, another hub gene, Nfkbia, was increased in LGG-treated colonoids. NFKBIA is the gene coding for IκBα, and the latter was one of the inhibitors of NF-κB. To investigate the role of Nfkbia/IκBα in intestinal inflammation, Mikuda et al. [35] generated a mouse model with IκBα specifically knocked out in IEC, and they detected a significant loss of Paneth cells and Krt15+ stem cells in the IκBα IEC-KO mice. Moreover, IκBα-deficient intestinal organoids presented a strong apoptotic response to a TNF-α or INF-γ treatment [35]. They indicated that a NF-κB activation caused by a Nfkbia/IκBα deletion triggered proapoptotic IECs. Therefore, Nfkbia/IκBα may be a target of the NF-κB-signaling pathway to maintain intestinal homeostasis.

However, this study has several limitations. First, the 3D spheroidal architecture of a colonoid is an obstacle for a microbe to access the apical side of colon epithelial cells. The Toll-like receptor (TLR) and other pattern recognition receptors vary, when comparing the apical and basal surfaces of cells. Therefore, the measurement of the barrier function is indirect in a 3D colonoid model. To solve the problem, the conversion of 3D colonoids into 2D monolayer colonoids is required in further studies. Second, although a prophylactic consumption of LGG decreased the sepsis mortality observed in this research, few studies have indicated the potential risk of bacteremia after a probiotic administration [36]. Consequently, further research is still required concerning the selection of the proper species of Lactobacillus, determining the appropriate amount of LGG, and finding the appropriate time to deliver the bacteria, whether before or after disease is presented.

## 5. Conclusions

Our results revealed that *LGG* promoted the recovery of the colon epithelial structure under sepsis and a TNF-α treatment. Based on a 3D colonoid model and RNA-seq, multi-pathways and multi-targets that play a role in ISC regeneration illustrated the therapeutic effect of *LGG* in sepsis. Moreover, *Tnfaip3* and *Nfkbia* may be used as two potential biomarkers for *LGG* in treating intestinal inflammation, and our research provides a new perspective on the therapeutic effect of *LGG.*

## Figures and Tables

**Table 1:** Primer sequences for qRT-PCR.

Target Genes	Primer Sense (5′-3′)	Primer Antisense (5′-3′)
*Lgr*5	CCTACTCGAAGACTTACCCAGT	GCATTGGGGTGAATGATAGCA
*Ascl*2	AAGCACACCTTGACTGGTACG	AAGTGGACGTTTGCACCTTCA
*Olfm*4	CAGCCACTTTCCAATTTCACTG	GCTGGACATACTCCTTCACCTTA
*Lyz*1	GAGACCGAAGCACCGACTATG	CGGTTTTGACATTGTGTTCGC
*Defa*6	CCTTCCAGGTCCAGGCTGAT	TGAGAAGTGGTCATCAGGCAC
*Tnfaip3*	ATGCACCGATACACACTGGA	GCGTGTGTCTGTTTCCTTGA
*Nfkbia*	TGAAGGACGAGGAGTACGAGC	TTCGTGGATGATTGCCAAGTG
*GAPDH*	ATGGTGAAGGTCGGTGTGAA	TGGAAGATGGTGATGGGCTT

## Data Availability

Data are available upon request.

## Abbreviations

ISC	intestinal stem cells
*LGG*	*Lactobacillus rhamnosus GG*
RNA-seq	RNA-sequencing
CLP	cecum ligation and puncture
GO	Gene Ontology
KEGG	Kyoto Encyclopedia of Genes and Genomes
DEGs	Differentially-expressed genes
PPI	Protein–protein interaction
